# Rapid Review on the Associations of Social and Geographical Isolation and Intimate Partner Violence: Implications for the Ongoing COVID-19 Pandemic

**DOI:** 10.3389/fpsyt.2021.578150

**Published:** 2021-04-13

**Authors:** Amera Mojahed, Stephanie Brym, Helene Hense, Bianca Grafe, Cornelia Helfferich, Jutta Lindert, Susan Garthus-Niegel

**Affiliations:** ^1^Institute and Policlinic of Occupational and Social Medicine, Faculty of Medicine, Technische Universität Dresden, Dresden, Germany; ^2^Center for Evidence-Based Healthcare, University Hospital Carl Gustav Carus and Carl Gustav Carus Faculty of Medicine, Technische Universität Dresden, Dresden, Germany; ^3^Social Science Research Institute for Gender Issues (SoFFI F.), Protestant University of Applied Sciences, Freiburg, Germany; ^4^University of Applied Sciences, Emden/Leer, Emden, Germany; ^5^Women's Research Center, Brandeis University, Waltham, MA, United States; ^6^Department of Medicine, Faculty of Medicine, Medical School Hamburg, Hamburg, Germany; ^7^Department of Child Health and Development, Norwegian Institute of Public Health, Oslo, Norway

**Keywords:** intimate partner violence, social isolation, geographical isolation, association, COVID-19, pandemic, rapid review

## Abstract

While the COVID-19 pandemic forced millions of people to stay home and minimize their social contacts, newspaper reports worldwide raised concerns as they reported an increasing rate of intimate partner violence (IPV). One link of the measures enforced to control the pandemic to IPV might be a possible side effect of those measures, namely social and geographical isolation. As there was no scientific data investigating the association of IPV and social and geographical isolation in the context of epidemics or pandemics at the time of conducting this rapid review, we aimed at investigating a broader range of contexts of social as well as geographical isolation and its association with IPV to draw conclusions regarding the COVID-19 pandemic. We searched Embase, PubMed, PsycINFO, and Web of Science (core collection). A research strategy was developed and observational studies were included if they considered men and/or women, estimates of social and geographical isolation, and IPV as a primary outcome. Of the 526 identified studies, 11 were included in this review. The included studies involved 15,695 women and were conducted in the USA, Sweden, Ethiopia, Egypt, Spain, and Turkey. Indicators of social isolation such as lack of social, emotional, or informational support or the frequency and quality of social contacts were narratively assessed. Geographical isolation was primarily assessed by physical distance to the next town or support service. Both social and geographic isolation were found to be associated with an increased risk of IPV. Recommendations made by the individual studies include the following: (a) improving access to social networks outside the victims' own group, (b) improving their economic circumstances, (c) asserting the responsibility for those in contact with the victims, and (d) increasing the focus on access to preventive services and programs need to be taken into account. Therefore, considering the particular infrastructure and legislation of the countries affected by the pandemic, policies need to ensure constant access to shelters and other help services and increase awareness for IPV in the society. In addition, future studies are warranted to assess prevalence rates and risk factors of IPV during the COVID-19 pandemic.

## Introduction

The COVID-19 outbreak, declared as a pandemic in March 2020 by the World Health organization [WHO] (1), forced several countries worldwide to impose strict measures to fight the outbreak of the virus. To contain infections, millions of people were forced to stay at home and minimize their social contacts. While physical and social distancing are effective measures to control the virus (2, 3), they showed negative impacts in other domains of public health. The resulting social isolation of such measures can be a major stressor that can contribute to widespread emotional distress, several psychological perturbations, and mood disturbances such as boredom, stress, depression, insomnia, irritability, anger, and frustration (4, 5). Possible distress within relationships with family and friends is also expected (6). Reports of newspapers and news agencies in several countries around the world reported an increasing rate of domestic violence among intimate partners (i.e., intimate partner violence (IPV) and against children, as well as an expected rise in femicide cases, child marriages, and genital mutilation in children since the implementation of the lockdown measures (7–11).

### Social and Geographical Isolation and IPV

IPV refers to any behavior within an intimate relationship that causes physical, psychological, and/or sexual harm to those in former or current relationships (12). Types of behavior could include: (A) acts of physical violence, such as slapping, hitting, kicking, and beating; (B) sexual violence, including forced sexual intercourse and other forms of sexual coercion; (C) emotional (psychological) abuse, such as insults, belittling, constant humiliation, intimidation (e.g., destroying things), threats of harm, threats to take away children; (D) controlling behavior, including isolating a person from family and friends, monitoring their movements, and restricting access to financial resources, employment, education, or medical care (12). IPV can happen to anyone, regardless of any gender specifications, and in any form of intimate relations (13). However, it is the most common form of violence against women, and approximately one in three women worldwide has experienced violence by an intimate partner during her lifetime (14).

Among the many factors that could contribute and affect the experience of IPV, isolation is a key concept for understanding IPV in various contexts (15). There are different understandings of social isolation, but with regard to the present study we refer to social isolation as a “lack of contact or of sustained interaction with individuals and institutions that represent mainstream society” (16) (p. 60). Social isolation is often measured by the type and extent of social support (17). In the case of IPV, for example, social support from individuals outside the intimate relationship has been recognized as an important protective factor and moderator of the effect of IPV on many physical and mental health outcomes (18, 19). In fact, it was suggested that the likelihood of violence against women decreases as the amount of social support available to them increases (20) and vice versa (21). Women who have friends or family members available for support seem therefore less socially isolated and thus in turn better protected from victimization at the hands of their partner than women without such support systems (22, 23). In addition, social isolation plays a major role in creating the structural dislocation of minorities and marginalized populations and the differential distribution of resources (i.e., social capital), which in turn could directly increase the risk for IPV victimization for individuals who face overlapping social discriminations due to their race, gender, class, etc. (13, 24, 25). Furthermore, geographical isolation can be defined by distance to resources like neighbors, friends, police stations, hospitals, or the nearest village or town (26). Such remoteness, which for instance can be found in rural areas, may also imply sociocultural and psychological isolation (27), thereby accentuating social isolation. Hence, social as well as geographical isolation could have implications for intensifying the hidden nature of IPV itself and undermine efforts to both seek and provide help (15).

The global pandemic and its consequences like lockdowns of entire nations represent a novel situation in several countries. Reports show the urgency to take a closer look at associations of IPV and the measures to control the pandemic (28, 29). One possible link might be a side effect of the imposed physical and social distancing (30). These preventative restrictions foster isolation and may result in victims of IPV being trapped at home with the perpetrators (12, 30). Apart from that, availability of social support systems such as family and friends might be limited; in addition, closed shelters and limited accessibility of protection services could make it more difficult for survivors to escape from their perpetrator (11, 30). Studies investigating the prevalence and possible underlying factors of IPV like social and geographical isolation during the COVID-19 pandemic are still inconclusive (31), and drawing conclusions from comparable situations in the past is limited. We found it most appropriate to conduct this rapid review which aims at investigating a broader range of pre-pandemic contexts of social and geographical isolation and their associations with IPV, as well as providing reliable, preliminary knowledge of their potential impact during the COVID-19 pandemic^1^. When investigating the association of IPV and social or geographical isolation, the bidirectional nature should be taken into consideration. On the one hand, studies have found that isolation is one of several negative outcomes of IPV (32). This association can be found in terms of coercive control, which implicates that social isolation can be caused by IPV through controlling several aspects of the victim's everyday life, such as limiting social contacts or access to professional help (33). On the other hand, studies investigated IPV against women found that many victims experienced physical and emotional aspects of IPV as a consequence of being forced into isolation by the perpetrator, suggesting that IPV could be a possible outcome of social and geographical isolation (34).

## Materials and Methods

Considering the necessity of addressing the issue of IPV in the context of the ongoing pandemic and in order to present relevant knowledge in a timely manner, we conducted this rapid review following the Cochrane guidelines for rapid reviews (35–37).

### Search Strategy

Research articles were primarily obtained through searches which were carried out in the following databases: Embase, PubMed, PsycINFO, and Web of Science (core collection). We used a combination of terms relating to IPV and social and geographical isolation, such as quarantine or social distancing as well as pandemics and epidemics. Separate searches for each primary database combined Medical Subject Subheadings (MeSH) terms and key text words with the Boolean operators (AND) and (OR), accordingly. The last date of the search considered for this review was on the 23rd of May, 2020 and was not restricted to any date range. The full list of search terms for PubMed can be found in the Appendix.

### Eligibility Criteria

For studies to be included in this review, we rigorously followed our population, intervention, comparison, and outcomes (PICOS) scheme. The target population were men and/or women in intimate relationships. The intervention was limited to the exposure to social and geographical isolation, as well as epidemics/pandemics. No comparators were considered. We considered IPV to be the only primary outcome for this review. We excluded any studies, which did not clearly report perpetrators as intimate partners or victims (e.g., children) for two main reasons. One was to keep the definition of our outcome clear and consistent throughout our review. The second reason was to reduce the possibility of including studies, which did not utilize adequate statistical models to disentangle the results (e.g., subgroup analyses for perpetrators other than intimate partners). Only empirical quantitative studies such as cohort, case-control, and cross-sectional studies were included, with qualitative studies being excluded. We originally planned to include only articles published in English and German, but we diverged from the protocol and considered articles published in Spanish for inclusion as well, since these languages are spoken by the authors.

### Data Collection Process

In order to conduct this rapid review, we used abbreviated systematic review methods and applied the following methodological shortcuts according to the Cochrane guidelines for rapid reviews: There was no dual abstract, dual full-text screening, dual data extraction, or dual assessment of risk of bias. All studies collected through the database searches were imported into the web-based, systematic review tool Rayyan QCRI (38). The identified titles and abstracts were then divided and screened; one reviewer (A. M.) screened titles and abstracts of studies identified by the search on PubMed, the other reviewer (H. H.) screened the ones identified by the search on Embase and PsycINFO. In case any of the reviewers were unsure whether titles and abstracts complied with the eligibility criteria, a second reviewer (S. B.) was consulted.

Full texts were then reviewed independently by the same reviewers (A. M.) and (H. H.) against the same inclusion and exclusion criteria as above. In case of uncertainties, a second reviewer (S. B.) was consulted. All studies that were accepted based on the full text screening were retained for data extraction. A data extraction form was developed where (S. B.) and (H. H.) then extracted data from each of the included studies. Extracted data included: author and year of publication, country, sample size, IPV prevalence estimates, type of isolation or its indicators, type of IPV (physical, sexual, psychological, and social), effect measures, as well as any recommendations made by the authors in the light of their findings.

### Risk of Bias (Quality) Assessment

Originally, we decided that the use of quality assessment tools was not feasible, due to the time constraints in conducting a rapid review. However, we diverged from the protocol and assessed the risk of bias of the included studies. According to the Cochrane guidelines for rapid reviews (37), the risk of bias should be limited to be rated by one reviewer (A. M.), with full verification of all judgements by a second reviewer (H. H.). We evaluated the overall risk of bias for each included study as “low,” “high,” or “unclear.” We followed the example used by Romero Starke et al. (39), and considering the criteria described by SIGN (40) and CASP (41). Items of the checklist were modified accordingly to suit the purpose of this review:

#### Recruitment Procedure

Adequate recruitment methods should be insured, such as randomized sampling. The response rate should be 50% or more, if not achieved, a non-participation analysis should take place. Studies that yielded high risk in this domain (i.e., studies that utilized convenience and clinical-populations) scored high risk in the overall assessment. For cohort studies, if the loss to follow-up was below 20% and there was no substantial difference between the comparison groups, this domain should be rated as low. Similarly, for a case-control study, both cases and control subjects should have a response of 50% or more, if this number was not achieved, non-participation analysis should be performed where substantial differential selection of cases and controls should be excluded. For cross-sectional designs, adequacy of randomization and inclusion criteria for participation, and an acceptable response rate to be 50% or more should be presented for this domain to be considered as low risk.

#### Exposure Definition and Measurement

The exposure should be defined as social and/or geographical isolation. Both or any other terms, such as social support, living in rural areas, etc., which fall under social or geographical isolation should be accurately stated and measured for this domain to be considered as low risk.

#### Outcome

The outcome should be defined as intimate partner violence (IPV). Other terms used for violence among intimate partners, e.g., domestic/family violence were considered to be high risk, because it would mean that other members of the family (father, brother, mother in-law, etc.) may have been co-perpetrators, and that is not what we aimed to measure. Nevertheless, if these terms were used, other indications of spousal/intimate violence should have been reported. IPV should be assessed with standardized validated IPV victimization tools, including self-report questionnaires.

#### Confounding

A list of potential confounders had to be given, such as age, location, region, years of education, socioeconomic status.

#### Analysis Methods

Studies had to include one of the following effect measures to assess associations of social and/or geographical isolation and IPV: Odds ratios (OR), correlations (r), differences between groups (d), or regression coefficients (B or beta). Also, adequate statistical models had to be used to reduce bias and control for confounding (e.g., standardization, adjustment in multivariate model, stratification, etc.) for this domain to be considered as having a low risk of bias.

#### Funding

The sources of funding and the involvement of the funding body in the research were assessed in this domain. This domain should be rated as having low risk, if a study was funded by a non-profit organization(s) and it was not affected by sponsors. If there was any participation in the data analysis or the study was probably affected by the sponsoring organization, the domain should be considered as high risk.

#### Conflict of Interest

Authors should report not having a conflict of interest for this domain to be rated as having a low risk.

#### Overall Assessment of Risk of Bias

We considered the first five domains (i.e., Recruitment Procedure to Analysis Methods) as major domains, while Funding and Conflict of Interest were considered as minor domains. We defined the overall scoring rules for the assessment of risk of bias for each study as high risk if any of the major domains was rated as “high risk” or “unclear risk.”

### Data Synthesis

We synthesized results narratively and in tabular form. Because of the heterogeneity of available primary studies, we did not consider conducting any quantitative analyses for this review.

## Results

### Description of Studies

**Figure 1:**
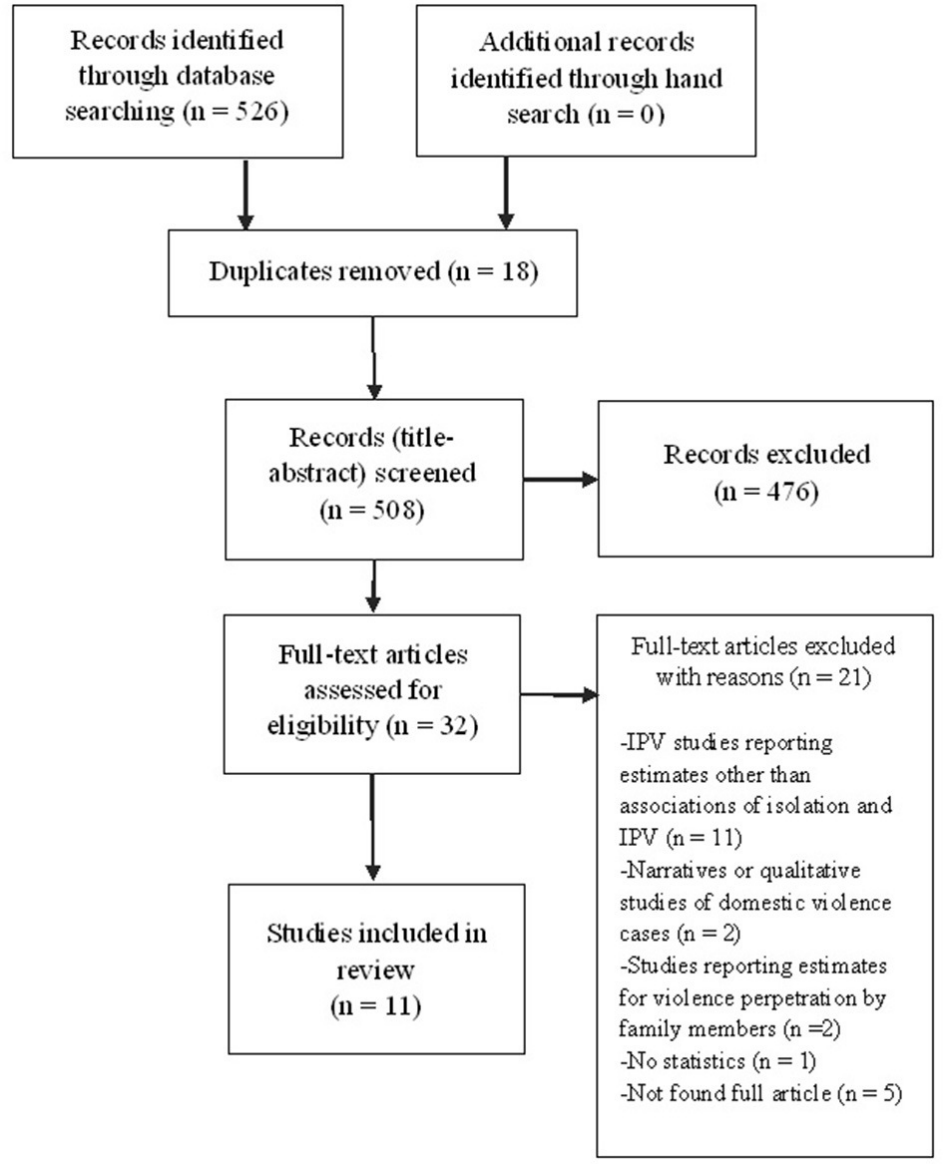
PRISMA flow chart.

The database search yielded 526 citations published between 1989 and 2020 (Figure 1). Articles were excluded based on information in the title and abstract. The full texts of potentially relevant articles were obtained for further assessment.

### Characteristics of Included Studies

Our searches identified 11 relevant studies (15, 42–51) (Table 1). Of these, nine studies were cross-sectional (42, 43, 45–51), one was longitudinal (15), and one comprised comparative case studies (44). They were published in English (*n* = 10) and Spanish (*n* = 1). The included studies involved 15,695 women. Six of the included studies were conducted in the USA (15, 42, 44, 45, 47, 48), followed by one study in Sweden (46), Ethiopia (43), Egypt (50), Spain (49), and Turkey (51), respectively. All of the included studies investigated violence against women where the sole perpetrator was their current or former male intimate partner. No study with men as victims was identified. Two terms were used to describe the violence, i.e., IPV (*n* = 8), and Domestic Violence (DV) (*n* = 3).

**Table 1:** Characteristics of included studies.

**Study id country**		**Setting**	**Study design**	**Sample size prevalence of IPV**	**Population's characteristics**		
1	Fernbrant et al. (46) Sweden	Population-based	Cross-sectional	804 Lifetime: 22.1% (emotional: 15.9%, physical/sexual: 14.8%) By previous partner: 20.5 % (emotional: 14.3%, physical/sexual: 13.9%) By current partner: 6.7% (emotional: 6.1%, physical/sexual:2.4%)	Thai women residing in Sweden (since 2006) *Age*: range: 18–64 years *Marital status*: 85.4% married/cohabiting *Occupation*: 39.3% employed *Education*: 0–9 years: 52.1%, 10 or more years: 47.9% *Self-indicated social isolation*: 39.9% *Self-rated mental health*: poor: 19.2%, good: 80.2%		
2	Lanier and Maume (15) USA	Populatio*n*-based	Longitudinal	4,914 Count of the number of times the female partner was the victim of physical violence in the past year, ranging from 0 to 4 (where four indicates four or more incidents):	Men or women married or cohabiting with an opposite sex partner (couples) Part of the NSFH (National Survey of Families and Households), waves 1 and 2		
				Non-metro counties: M = 0.09 (SD = 0.48)		Non-metro counties *N* = 4,006	Metro counties *N* = 4,636
				Metro: M = 0.10 (SD = 0.48) *number of incidents in past year (one or more):* non-metro: 5.08%, metro: 5.87%	*Age*	M = 41.41 (SD = 16.66)	M = 39.53 (SD = 14.02)
					*Ethnicity*	Black: 11.42%, Hispanic: 3.86%, White: 84.72%	Black: 15.1%, Hispanic: 6.57%, White: 78.33%
					*Income-to-needs ratio*	M = 4.99 (SD = 4.95)	M = 3.82 (SD = 3.18)
					*number of kids <18*	M = 1.09 (SD = 1.25)	M = 1.07 (SD = 1.28)
					(Demographics refer to whole sample of *N* = 8,642)		
3	Farris and Fenaughty (45) USA	Population-based	Cross-sectional	262 At least one incident of physical violence: 38.2%	Female drug users (most commonly used substance: smokable cocaine), part of a larger study, investigating HIV sexual risk behaviors, street-recruited *Age*: >18 years old/mean age: 37.6 y (SD = 6.8) *Ethnicity*: Caucasian: 41%, Alaska Natives/ American Indians: 32%, African American: 21% *Education*: Less than high school: 32.4%, high school graduation or GED: 36.3%, more than high school: 31.3% *Monthly income from all sources*: M = $1144 (SD = $2358) *Monthly legal income*: M = $557 (SD = $727) *Social class (self-reported):* Upper class: 3.5%, middle class: 22.4%, working class: 25.9%, lower class: 30.1%, truly needy: 18.1% *Number of children at home*: M = 0.6 (SD = 1.5)		
4	Peek-Asa et al. (48) USA	Clinic-based	Cross- sectional	1,478 Overall: 16.1% (physical/sexual: 12.5%, battering: 9%) Urban towns: 15.5% Large rural towns: 13.5% Small rural towns: 22.5% Isolated rural areas: 17.9%	Women who attended for elective abortion at a clinic, Iowa residents No table for demographic information		
5	Bosch and Schumm (42) USA	Populatio*n*-based	Cross-sectional	56 100%	Women who experienced an abusive relationship *Age*: *M* = 40.5 years (SD = 8.6), range: 22–63 years *Ethnicity*: Caucasian: 84%, Native-American: 14%, African American: 2% *Marital Status*: 80% married during abusive relationship (at time of interview, only one woman was still married to formerly abusive husband) *Education:* Less than high school: 5%, college degree: 14%, some college training: 40% additional vocational training: 9% *Occupation*: 49% working part-time, 38% working full-time during abusive relationship *Mean annual household income* (during abusive relationship): *M* = $34250, (SD = $29319) 36% receiving consistent monetary support 90% with minor children (average of 3.5 children, SD = 1.5)		
6	Chernet and Cherie (43) Ethiopia	Populatio*n*-based	Cross-sectional	4,714 30%	Ever-married women, survey of households *Age:* range 15–49 years (15–19: 22.7%, 20–24: 19.4%, 25–29: 20.0%, 30–34: 15.2%, 35–39: 10.8%, 40–44: 6.9%, 45–59: 5.0%) *Marital Status*: 71.3% married, 56.4% divorced, 73.5% widowed *Education*: Uneducated: 49.0%, primary: 34.2%, secondary: 11.4%, higher: 5.4% *Wealth index*: Poor: 44.7%, middle: 14.1%, rich: 41.2% 74.3% living in rural areas, 25.7% in urban areas		
7	Seedhom (50) Egypt	Populatio*n*-based	Cross-sectional	1,502 physical violence: 30.3% sexual violence: 7.5% sexual and physical violence: 31.6% emotional violence: 49.3% All forms of violence: 60.4%	Currently or formerly married women, systematic random sample from an Egyptian city *Age*: range 18–65 years (18–29: 21.6%, 30–44: 41.4%, 45–65: 37.0%) *Marital status*: 86% married, 14% divorced/ separated/ widowed *Education*: Illiterate: 40.2%, read and write: 35.3%, below University level: 14.9% University level or above: 9.6% *Occupation*: 77.3% housewife, 22.6% employed		
8	Plazaola-Castaño et al. (49) Spain	Clinic-based	Cross-sectional	1,402 any type of violence during lifetime: 32.0% physical violence and sometimes psychological: 7.0% psychological violence: 14.0% psychological and sexual violence: 2.5% all three types of violence: 6.0%	Women who seeked help at a primary care center *Age*: *M* = 38.83 years (SD = 11.15), range: 18–65 *Marital status*: 62.9% married, 25.6% single, 11.5% separated/ divorced/ widowed *Education*: University degree: 34.7%, high school: 23.7%, middle school: 37.9%, no education: 3.7% *Occupation*: 35.3% housewives, 51.0% employed, 13.7% student/ unemployed *Monthly income*: > 1,200€: 36.0%, 900–1,200€: 23.5%, 600–900: 25.5%, <600: 15.0% *Number of children*: none: 29.7%, one: 20.5%, two: 33.3%, three or more: 16.4%		
9	Coohey (44) USA (Iowa)	Recruited from parent groups in public schools, social service agencies, day care centers + libraries in Chicago	Comparative case studies	143 No prevalence estimates were provided	Mothers with a current partner (40 severely assaulted battered mothers, 46 battered but not severely assaulted mothers, 57 not battered mothers) *Age*: *M* = 30.56 years *Ethnicity*: 33.49% African American, 35.46% Latina American, 33.7% Anglo-American *Marital status*: 47.53% married *Education*: *M* = 11.23 years 86.03% lived below 120% of the poverty line *Number of children*: *M* = 2.99		
10	Yanikkerem et al. (51) Turkey	Population-based	Cross- sectional	217 9.7% rural areas: 17.3% urban areas: 2.7%	Pregnant women living in certain areas (household survey) *Age*: 42.4% aged 25 years and younger, 57.6% older than 25 years *Marital status*: 82.5% married, 17.5% unmarried *Education*: elementary or less: 75.1%, more than elementary: 24.9% *Occupation*: 6.5% employed, 93.6% unemployed *Income status*: High to middle: 81.1%, low: 18.9% 52.1% attended clinic in urban areas, 47.9% in rural areas		
11	Levendosky et al. (47) USA (Michigan)	Populatio*n*-based	Cross-sectional	203 71.4%	Women in their last trimester of pregnancy *Age*: *M* = 25.3 years Ethnicity: 63% Caucasian, 25% African American, 5% Latina, 7% other ethnic *backgrounds* *Marital status*: 49% single + never married, 40% married, 11% separated/ divorced/ widowed *Education*: Less than high school: 16%, high school: 30%, post-high-school training: 41%, college: 13.5% *Monthly income*: Median = $1500, range: $0–$9500 *Number of children*: one or more: 57%		

### Quality of Included Studies

Seven studies scored high risk of bias (42, 44, 45, 47–49, 51), while four studies scored low risk of bias (15, 43, 46, 50). Table 2 summarizes the risk of bias assessment scores for the included studies.

**Table 2:** Risk of bias of included studies.

**Study ID**	**Bosch and Schumm (42)**	**Chernet and Cherie (43)**	**Coohey (44)**	**Farris and Fenaughty (45)**	**Fernbrant et al. (46)**	**Lanier and Maume (15)**	**Levendosky et al. (47)**	**Peek-Asa et al. (48)**	**Plazaola-Castaño et al. (49)**	**Seedhom (50)**	**Yanikkerem et al. (51)**
**MAJOR DOMAINS**											
Recruitment Procedure	High risk	Low risk	High risk	High risk	High risk	Low risk	Unclear risk	Unclear risk	High risk	Low risk	High risk
Exposure Definition and Measurement	Low risk	Low risk	Low risk	Low risk	Low risk	Low risk	Low risk	Low risk	Low risk	Low risk	Low risk
Outcome	Low risk	Low risk	Low risk	Low risk	Low risk	Low risk	Low risk	Low risk	High risk	Low risk	Low risk
Confounding	Low risk	Low risk	Low risk	Unclear risk	Low risk	Low risk	Low risk	Low risk	Unclear risk	Low risk	Low risk
Analysis Methods	Low risk	Low risk	Low risk	Low risk	Low risk	Low risk	Low risk	Low risk	High risk	Low risk	High risk
**MINOR DOMAINS**											
Funding	Unclear risk	Low risk	Unclear risk	Unclear risk	Unclear risk	Low risk	Unclear risk	Unclear risk	Unclear risk	Unclear risk	Unclear risk
Conflict of Interest	Unclear risk	Low risk	Unclear risk	Unclear risk	Unclear risk	Low risk	Unclear risk	Low risk	Unclear risk	Low risk	Unclear risk
Overall Assessment	High risk	Low risk	High risk	High risk	Low risk	Low risk	High risk	High risk	High risk	Low risk	High risk

### Associations of Social and Geographical Isolation and IPV

Two studies reported associations of social isolation and IPV (45, 46). In Farris & Fenaughty (45), social isolation was strongly correlated with physical and sexual IPV among female drug users. In another study, social isolation was reported among immigrant women as a predictor for physical, sexual, and psychological IPV (46). Both social and geographical isolation were reported in two of the included studies (15, 42). Social isolation was assessed in terms of lack of emotional and informational support and found to be a predictor for an increased risk of IPV among women, who were also geographically isolated. They were found to be living approximately 6 miles away from the closest town, 12 miles away from closest mental health center, and 78 miles away from closest shelter service (42). In Lanier & Maume (15), social isolation was assessed in terms of lack of social support. Variables such as lack of help received, interaction through socializing, and church participation were measured and found to be significantly associated with increased risk of IPV. The geographical isolation aspect was assessed according to the counties classification into metropolitan countries, if they were located in a metropolitan area and contained an urban population of 20,000 or more, or non-metropolitan counties, which are an approximation of the rural context. It was also combined with the disadvantage index (i.e., sum of relative presence of Black residents, poverty households, female-headed households, and the unemployed in the county), as well as the Gini index (i.e., a standard measure of income inequality ranging from 0 to 100, where 100 indicates perfect inequality). The model for respondents in non-metro counties indicated the likelihood of women experiencing IPV in the past year was reduced significantly as levels of help received increased. Other findings indicating that respondents living in metro counties with higher levels of income inequality also reported a greater degree of IPV. This was also true for respondents in metro counties with more minor children in the household.

Four studies investigated lack of social support as indicator for social isolation (44, 47, 49, 50). Coohey (44) found that mothers who were severely assaulted, had fewer friends, fewer contacts with their friends, fewer long-term friendships, and fewer friends who really listened to them than did the non-battered mothers and the battered mothers who were not severely assaulted. In another study, social isolation was assessed by measuring the quality of support among a network of pregnant battered women (47). However, correlations between the average severity of violence and the practical, emotional, and critical support were not found to be statistically significant. Yet, for battered women, the number of supporters in their network who were in an abusive relationship as well, was related to impaired emotional and critical support among these women. No further investigations were made regarding the association between this similarity of battered women and their supporters and IPV (47). In Plazaolo-Castaño et al. (49), women who reported having social support had a lowered probability of ever being abused than women who reported not having social support. Women who experienced abuse in the past and currently having social support had a lower probability of being abused again by a different partner than those who had no social support. Lack of social support was also investigated in Seedhom (50) and it was considered a predictor for physical, social, and emotional violence. Three studies investigated geographical isolation (43, 48, 51) and found it to be a risk factor for IPV. Chernet & Cherie (43), and Yanikkerem and colleagues (51) found that women living in rural areas were at significantly higher risk compared to women living in urban areas (Table 3).

**Table 3:** Findings of individual studies.

**Study id/country**		**Type of isolation**	**Type of IPV**	**Effect measures**	**Recommendations by authors**
1	Fernbrant et al. (46) Sweden	Social isolation	IPV (physical, sexual, psychological)	OR: 3.37 (1.82–6.24)	The role of social capital in increasing resilience against poor mental health for those living in abusive relationships indicates a need for supporting social structures that facilitate Thai women's' access to networks outside their own group.
2	Lanier and Maume (15) USA	Geographic isolation, social isolation (social support)	IPV (physical or sexual assault, threat of assault)	Non-metro counties/ metro counties (*N* = 1,781/*N* = 3,133): Help received: β = −0.218/0.060 (*p* = 0.19/0.374) Interaction—socializing: β = 0.053/−0.004 (*p* = 0.20/0.919) Interaction—church β = −0.040/−0.022 (*p* = 0.518/0.617) Interaction—participation β = 0.004/−0.004 (*p* = 0.923/0.882)	The study suggests that policies that work to increase the social networks of women living in rural areas may effectively decrease violence.
3	Farris and Fenaughty (45) USA	Social isolation	IPV (physical, sexual)	OR = 5.17 (2.62–10.19)	People who do have contact with this disadvantaged population have an added responsibility to provide immediate support and resources Intervention cannot be aimed singularly at social isolation, as isolation is likely to be tied closely to experiences of violence and drug use.
4	Peek-Asa et al. (48) USA	Geographic isolation	IPV (physical, sexual, psychological)	OR = 1.2 (0.7–2.1)	Increased focus on access to preventive services, including Domestic Violence Intervention Programs (DVIP) resources, is critically needed.
5	Bosch and Schumm (42) USA	Social and geographic isolation (~6 miles from closest town/grocery store, 12 miles from closest mental health center, 78 miles from closest shelter services)	IPV	***Previous/current abuse*** access to resources: *r* = −0.381^**^ emotional non-support: *r* = 0.355/0.360^**^ ***Abuse during relationship*** access to resources: β = 0.515^***^ emotional support: β = 0.423^***^ ***Abuse at time of interview*** informational support: β = −0.577^***^ (^**^p < 0.01, ^***^p < 0.001)	It is imperative that persons in the informal and formal networks take individual responsibility in holding abusers accountable for their abusive behaviors. Women should not be held totally responsible for tackling this societal issue on their own. Practitioners must work with the informal and formal networks within communities to provide information and advice, help women access resources, and hold abusers accountable.
6	Chernet and Cherie (43) Ethiopia	Geographic isolation (living rural as a predictor)	IPV (physical, sexual, emotional)	Living in rural, being poor, being divorced and being 25–39 years old are found to be significant predictors if IPV Urban area: OR = 0.66 (0.5353–0.8127)	Improving economic status of household and awareness creation for rural residents can be effective strategies to reduce IPV.
7	Seedhom (50) Egypt	Lacking social support; being separated/ widow/ divorced	IPV (physical, sexual, emotional)	Lifetime prevalence of IPV lower for women with social support: 18.4 vs. 16.6% (*p* < 0.002) *logistic regression (social support as predictor):* physical and social violence: β = 1.63, OR = 7.8 (3.12–14.60) emotional violence: β = 1.12, OR = 9.6 (4.20–20.40)	-
8	Plazaola-Castaño et al. (49) Spain	Lack of social support	IPV (physical, psychological and sexual)	Social support and abuse: OR = 0.11 (0.06–0.20) Social support and recurring abuse: OR = 0.14 (0.05–0.37)	Interventions should aim at re-establishing social networks of women experiencing abuse.
9	Coohey (44) USA (Iowa)	Lack of social networks and received support (family and friends)	Domestic violence	Association between being severely assaulted and social network/ support characteristics: Size of friendship network: *r* = −0.17^*^ Number of contacts with friends: *r* = −0.17^*^ Number of long-term friends: *r* = −0.23^*^ Number of friends who really listened: *r* = −0.22* (^*^p < 0.05)	As battered women were more likely to seek out support from family and friends than from professional helpers after a battering incident, interventions that include members of a woman's social network might be effective in keeping them and their children safe.
10	Yanikkerem et al. (51) Turkey	Geographic isolation (rural area)	Domestic violence against pregnant women	Women who lived rural area had experienced violence more than women who lived in urban areas (*p* < 0.05) Higher numbers of violence in women seeking prenatal clinics (139.3 vs. 199.8, *p* = 0.000)	–
11	Levendosky et al. (47) USA (Michigan)	Lack of social support (structural support, e.g., total number of supporters; functional support, e.g., emotional)	Domestic violence	Isolation assessed by measuring quality of support: correlation of average severity of violence and practical/emotional/ critical support: 0.08/ 0.00/−0.13 (not significant)	–

* *p < 0.05*,

^**^p < 0.01, ^***^p < 0.001.

### Recommendations Made by Individual Studies

As a summary of the recommendations made by the individual studies, Coohey (44) pointed out that battered women were more likely to seek out support from family and friends than from professional helpers. Besides, interventions should aim at re-establishing social networks of women experiencing abuse (49). It was also emphasized that interventions for women living in rural areas should not be limited to formal networks, but should also include informal (social) networks within the community in order to provide information and advice, help women access resources and hold abusers accountable (15, 42). These studies expressed how imperative it is that abusers are held accountable for their abusive behaviors. In the case of socially isolated migrant women, this focus should be applied to the social structures as a whole to improve women's access to networks outside their own group (46). Moreover, improving the economic status of rural households could be an effective strategy to reduce IPV (43). Apart from that, as isolation is also likely to be tied closely to experiences of violence and drug use for the disadvantaged population of abused female drug users, people who have contact with victims ought to provide immediate support and resources (45). Finally, Peek-Asa et al. (48) recommended increasing the focus on access to preventive services in the case of rural women, including Domestic Violence Intervention programs (DVIP) resources.

## Discussion

The objective of our rapid review was to investigate the associations between social and geographical isolation and IPV and their possible implications for the ongoing COVID-19 pandemic. In this rapid review, the literature search did not reveal any studies associated with social or geographical isolation in the context of epidemics or pandemics. This means that the applicability of our conclusions regarding the ongoing COVID-19 pandemic could be limited. However, as we already argued in the beginning, the ongoing pandemic represents a novel situation, it was therefore inevitable for us to consider pre-lockdown contexts as an approach to draw conclusions.

We found isolation, both social and geographical, was associated with IPV. Indicators of social isolation varied across studies. While two studies assessed social isolation directly (45, 46), there was a variety of approaches assessing social isolation indirectly among the other studies. Those approaches included assessing lack of social support (49, 50), lack of emotional or informational support (42), lack of practical, emotional, and critical support (47), number of friends or frequency of contacts (44), as well as membership in social networks and levels of social interaction (15). Having one of those indicators alone does not necessarily indicate being socially isolated, but when combined with other factors, such as unemployment, poverty, or drug use, they may provide an adequate indicator of social isolation (34). These findings are consistent with most recent studies which suggest that increasing feelings of isolation during the COVID-19 lockdown measures may cause abuse of alcohol, drugs, as well as increased anger and aggression, which may also lead to violence toward the self or others (52), such as one's intimate partner (53). Combined with isolation, experiencing economic problems caused by an ongoing lockdown can significantly contribute to the increase of stress in an already strenuous relationship, precipitating IPV episodes (54). Indeed, initial studies and reports indicate changes in the prevalence of IPV and the extent of injuries. For instance, latest figures imply either a decline or an increase in IPV cases in various countries. However, where there has been a reported decrease, it was in stark contrast to the severity of the injuries that have been presented (55). Thus, the current research evidence remains inconclusive, since there are few representative surveys and figures available. In any case, IPV interventions and the care of affected individuals and their children must be guaranteed even in times of an ongoing pandemic, where urgent adaptation of intervention and protection measures of IPV to these special conditions, as well as the timely announcement of corresponding help offers are of central importance.

### Implications for the Ongoing COVID-19 Pandemic

Many of the included studies have emphasized social support through the recommendations that they made in order to enhance the interventions and prevention of IPV in the context of isolation. Of these studies, some have expressed living in rural areas (i.e., being geographically isolated) could correlate with social isolation, which in turn could increase the risk of IPV victimization (15, 42). Such isolation could be very similar to our context of the COVID-19 pandemic, where physical entrapment of potential victims is seen due to the enforced quarantine and physical and social distancing rules. Furthermore, this remoteness or entrapment with emergency resources being limited, such as the closure of women's shelters and ambulatory and community referral sites during the pandemic, could render victims more vulnerable to IPV (56, 57). Even without isolation, access to information and support could be a difficult task for women in violent relationships. In times where personal freedom is restrained even more, digital means of communication such as m-health, social media, or telemedicine could play an important role in reducing the sense of isolation and entrapment the victims may suffer, and could facilitate better access to key workers (e.g., helplines, legal aid) and foster better support (11). The generalizability of how isolation and IPV are associated is limited due to the heterogeneous characteristics of the included study populations, like the fact that some studies were conducted in low and middle income countries such as Ethiopia (43), while others were conducted in high-income countries like Sweden (46). Some studies included very specific populations such as female drug users (45), women who attended for elective abortion (48), pregnant women (47, 51), and migrant women (46). Nevertheless, our results shed light on the possible increased likelihood for these populations to experience IPV under the COVID-19 pandemic circumstances. Therefore, the recommendations of those studies, such as improving access to social networks outside the victims' own group, improving their economic circumstances, asserting the responsibility for those in contact with the victims, and increasing the focus on access to preventive services and programs need to be taken into account. It is also very important for the governments around the globe to develop innovative strategies in order to ensure access to all the relevant information and the infrastructure in place, along with the required services, during this crisis situation. This is especially important for those being at most danger (i.e., women, children, elderly) (58). Moreover, the cross-sectional design of some of the included studies does not allow us to determine whether IPV consequently leads to isolation, especially social isolation, or whether isolation rather serves as cause of IPV. Nevertheless, findings in our review show that isolation is strongly associated with an increased risk of IPV. This could be applied to the context of this rapid review since isolation could be seen as a consequence of the physical and social distancing, as well as quarantine during this pandemic.

### Limitations

We conducted a rapid review due to the urgency of the topic and its implications for the ongoing COVID-19 pandemic. As a result, time constraints asked for an abbreviation of certain methodological steps of the review process. Since neither dual titles-abstract nor dual full-text screening were performed, relevant studies might have been missed and a certain selection bias might have been introduced. Only published studies with language restriction (i.e., English, German, and Spanish) were used, this could mean that some eligible studies may be missed, resulting in a selection bias. Upon our risk of bias assessment, seven studies were found to be of high risk. This could influence the quality of the rapid review in general, causing mainly reporting bias. Nevertheless, the present rapid review contains clear eligibility criteria. Our procedures, which were based on the guidance and training materials produced by Cochrane for rapid reviews make us assume that the overall conclusion was not affected by those limitations.

## Conclusions and Implications for Future Research

In this review, we aimed at identifying possible associations between social and geographical isolation and IPV to assess their potential impact during the ongoing COVID-19 pandemic. Overall, our narrative synthesis of the pre-pandemic data emphasized that isolation could be associated with experiencing IPV in the context of the current pandemic. Associated factors like limited access to formal and informal services as well as disruptions of social networks has affected millions of people during the pandemic due to quarantine, and physical and social distancing measures. Therefore, isolation circumstances should be seriously considered as an important factor regarding recommendations made by the individual studies for interventions and prevention of IPV. Policies need to make sure that alternative help services (e.g., messenger services, telemedicine) are accessible and dependable by victims of IPV who are affected by isolation with particular attention to reaching survivors safely while perpetrators are present and in ways that cannot be detected or traced. In addition, increasing awareness for IPV is essential so that people working in the informal or formal sector as well as family and friends in the immediate social network of IPV victims are sensitized to signs of violence.

Additionally, help systems in the countries included in the review differ widely. Therefore, conclusions of this review have to be adopted to fit the particular help systems, infrastructure, and legislation. Measures such as pharmacies establishing code words for victims to get help were established in Belgium, France, Spain, the Netherlands, and Germany. For example, in Germany, the national coalition of pharmacist organizations (Bundesvereinigung Deutscher Apothekerverbände e.V.), the national coalition of women's counseling services (Bundesverband Frauenberatungsstellen und Frauennotrufe [bff]), and the national helpline against violence against women (Hilfetelefon Gewalt gegen Frauen) started a national campaign. Nineteen thousand pharmacies are providing information about the national helpline since pharmacies belong to the very few places where women can access low threshold information regarding health and well-being during the pandemic. This campaign raises awareness for the possibility of 24/7 free and anonymous counseling. The national helpline is of key importance. It is free, available at all times, and it offers counseling for female victims, translation, information, and redirection to a local counseling service and/or shelter. While face-to-face counseling and admission to shelters has proven problematic during the pandemic, the website and phone service remain of vital importance and safe options during isolation. Also, the Fed, the Ministry for Family Affairs, Senior Citizen, Women and Youth in Germany started a cooperation with supermarkets, displaying information regarding help hotlines or services on posters and the back of receipts. To establish more conclusive evidence, a systematic review with meta-analysis is currently being performed by one of this study's co-authors (J. L^2^.)

## Data Availability Statement

The original contributions generated for this study are included in the article/Supplementary Material, further inquiries can be directed to the corresponding author/s.

## Author Contributions

AM, SG-N, SB, and HH designed and conceptualized the present study. AM, SB, and HH conducted manuscript screening and data extraction. AM wrote the first draft of the manuscript. SG-N supervised data extraction and drafting of the manuscript. AM, SB, HH, BG, CH, JL, and SG-N contributed to the analysis and interpretation. AM, HH, and SG-N contributed to the manuscript revision. All authors read and approved the submitted version.

## Conflict of Interest

The authors declare that the research was conducted in the absence of any commercial or financial relationships that could be construed as a potential conflict of interest.

## Footnotes

^1^https://www.crd.york.ac.uk/prospero/display_record.php?RecordID=185917.

^2^https://www.crd.york.ac.uk/prospero/display_record.php?ID=CRD42020186517.
