# Supplementary material for: Rapid Review on the Associations of Social and Geographical Isolation and Intimate Partner Violence: Implications for the Ongoing COVID-19 Pandemic
Source: Front Psychiatry. 2021 Apr 13;12:578150. doi: 10.3389/fpsyt.2021.578150 (PMC8076499; doi:10.3389/fpsyt.2021.578150)
Supplement: Supplementary file 1 [file Table_1.DOCX]

Supplementary Material

# Appendix

Table 1: Search terms for PubMed

| #1 | (((((((((intimate partner violence[MeSH Terms]) OR domestic violence[MeSH Terms]) OR spousal violence[MeSH Terms]) OR dating violence[MeSH Terms]) OR relationship violence[MeSH Terms]) OR couple violence[MeSH Terms]) OR marital violence[MeSH Terms]) OR physical violence[MeSH Terms]) OR emotional violence[MeSH Terms]) OR psychological violence[MeSH Terms]) OR sexual violence[MeSH Terms] |
| --- | --- |
| #2 | ((((((pandemic[MeSH Terms]) OR epidemic[MeSH Terms]) OR social isolation[MeSH Terms]) OR rural areas[MeSH Terms]) OR geographical isolation[Text Word]) OR quarantine[Text Word]) OR social distancing[Text Word] |
| #3 | ((adult[MeSH Terms]) OR aged[MeSH Terms]) OR young adult[MeSH Terms] |
| #4 | (((((((longitudinal[Text Word]) OR prospective[Text Word]) OR cohort studies[MeSH Terms]) OR cohort study[MeSH Terms]) OR retrospective[Text Word]) OR cases[Text Word]) OR controls[Text Word]) OR cross sectional[Text Word] |
